# Sphingolipids: Effectors and Achilles Heals in Viral Infections?

**DOI:** 10.3390/cells10092175

**Published:** 2021-08-24

**Authors:** Sibylle Schneider-Schaulies, Fabian Schumacher, Dominik Wigger, Marie Schöl, Trushnal Waghmare, Jan Schlegel, Jürgen Seibel, Burkhard Kleuser

**Affiliations:** 1 Institute for Virology and Immunobiology, University of Wuerzburg, 97078 Würzburg, Germany; s-s-s@vim.uni-wuerzburg.de (S.S.-S.); marie.schoel@uni-wuerzburg.de (M.S.); trushnal.waghmare@uni-wuerzburg.de (T.W.); 2 Institute of Pharmacy, Pharmacology and Toxicology, Freie Universität Berlin, 14195 Berlin, Germany; Fabian.schumacher@fu-berlin.de (F.S.); Dominik.Wigger@fu-berlin.de (D.W.); 3 Department for Biotechnology and Biophysics, University of Wuerzburg, 97074 Würzburg, Germany; jan.schlegel@uni-wuerzburg.de; 4 Department for Organic Chemistry, University of Wuerzburg, 97074 Würzburg, Germany; seibel@chemie.uni-wuerzburg.de

**Keywords:** glycosphingolipids, ceramides, sphingosine 1-phosphate, sphingomyelinase, HIV, SARS-CoV-2, measles

## Abstract

As viruses are obligatory intracellular parasites, any step during their life cycle strictly depends on successful interaction with their particular host cells. In particular, their interaction with cellular membranes is of crucial importance for most steps in the viral replication cycle. Such interactions are initiated by uptake of viral particles and subsequent trafficking to intracellular compartments to access their replication compartments which provide a spatially confined environment concentrating viral and cellular components, and subsequently, employ cellular membranes for assembly and exit of viral progeny. The ability of viruses to actively modulate lipid composition such as sphingolipids (SLs) is essential for successful completion of the viral life cycle. In addition to their structural and biophysical properties of cellular membranes, some sphingolipid (SL) species are bioactive and as such, take part in cellular signaling processes involved in regulating viral replication. It is especially due to the progress made in tools to study accumulation and dynamics of SLs, which visualize their compartmentalization and identify interaction partners at a cellular level, as well as the availability of genetic knockout systems, that the role of particular SL species in the viral replication process can be analyzed and, most importantly, be explored as targets for therapeutic intervention.

## 1. Introduction

Sphingolipids (SLs) are highly abundant components of cellular membranes and as such, are essentially involved in their biophysical and signaling properties. A complex metabolic network consisting of enzymes catalyzing their synthesis, modification (phosphorylation, glycosylation) and breakdown regulates accumulation of sphingolipid species and thereby the sphingolipid pool at rheostat conditions, and this can undergo substantial changes in response to metabolic and external challenges. This has been excellently reviewed [1,2,3] and will therefore just be briefly re-iterated below. Depending on the length of their fatty acid chains and their degree of saturation, SL species have a strong impact on biophysical membrane parameters such as fluidity or rigidity and curvature, and on interaction with membrane proteins and/or cytoskeletal components, and membrane compartmentalization. This coins their ability to promote the formation and activity of signaling platforms in a dynamic and spatiotemporally regulated manner [4,5,6,7]. In addition to structurally supporting the interaction of receptors with their membrane proximal signalosome components, certain SL species (especially ceramides, ceramide-1-phosphate, sphingosine and sphingosine 1-phosphate) act as highly potent signaling molecules themselves, regulating, for instance, cellular apoptosis and autophagy, or activation and survival, respectively [1,2,8,9,10,11,12]. Therefore, dynamic alterations of the SL pool and its subcellular compartmentalization not surprisingly have a substantial impact on most cellular processes determining viability and responsiveness. Supporting their decisive role also in clinical terms, alterations in SL biosynthesis or accumulation are of crucial importance in the pathophysiology in severe diseases including lysosomal storage diseases and cancer, and pharmacological interference with SL metabolism has already been proven as an effective target for treatment of major depression, cancer and inflammation [10,13,14,15,16].

Lack of suitable reagents for detection and fixation or protocols for quantification of SL species have long hampered detailed studies in SL biology at a cellular level, while on an organismic level, ubiquitous genetic ablation of SL modifying enzymes in mice, in some cases reliably reproducing disease processes in humans, precluded detailed analysis on the pathophysiological role of individual compartments. With the advent of bio-orthogonally functionalized SLs, important progress has been made in their detection and recording their trafficking at high resolution, identifying associated protein complexes, and, in combination with compartment-specific targeting of SL modifying enzymes, determining topology and biological consequences of their metabolism [17,18,19,20,21,22]. Mass spectrometry is the favored analytical technology for SL analysis. Thus, this technique has the capability to both acquire sensitive and quantitative measurements and to unravel the molecular intricacies of SL species. At present, mass spectrometric analysis of lipid extracts, mainly by infusion electrospray ionization, is utilized to determine sensitive and quantitative information of specific SLs in biological processes. As a complementary method, mass spectrometry imaging, albeit less quantitative and less specific, provides information of spatial SL distribution in tissues.

These techniques are being exploited in the context of general SL biosynthesis and turnover, which, due to its central role in cell physiology, directly translates into regulation of cellular processes, and therefore has been targeted for therapeutic intervention as referred to above. The last two decades have seen an increasing amount of studies addressing the role of the SL pathway in infections where their turnover has been found to regulate the disease process both at the level of the pathogens’ life cycle and efficiency of immune control. Rather than discussing that process in bacterial infections (which has been excellently reviewed [23,24,25,26]), this review focuses on the role of sphingolipid metabolites in viral infections. Especially the importance of the SLs in governing essential steps in virus–host cell interaction is described.

## 2. Sphingolipid Metabolism

**Figure 1:**
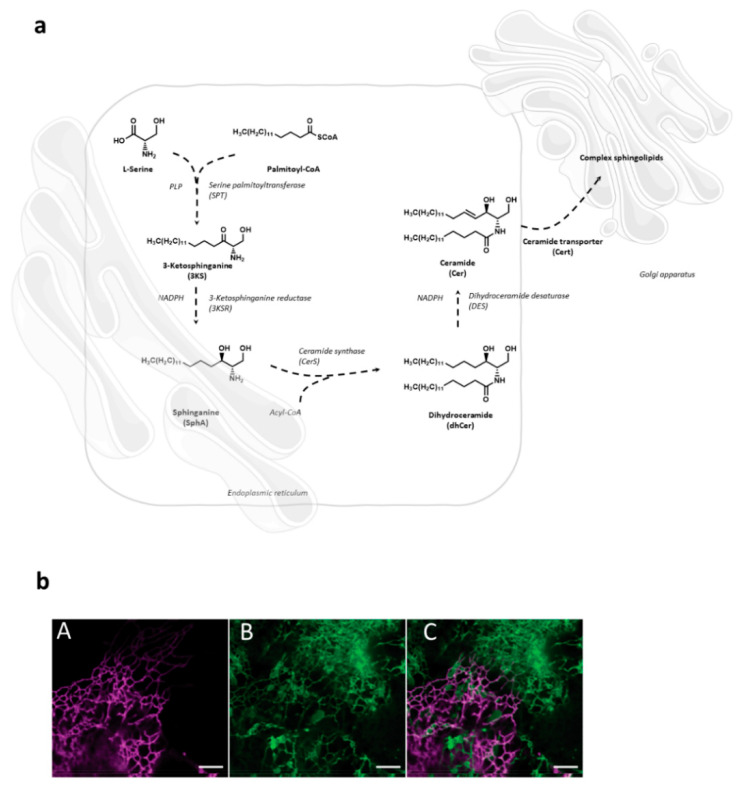
(**a**) Schematic overview of the SL de novo synthesis located in the ER. A cell-free assay utilizing rat liver microsomes containing all the enzymes necessary for bottom-up synthesis of ceramides can be used to study the entire de novo pathway via mass spectrometry [28]. The pyridoxal 5′-phosphate (PLP)-dependent enzyme serine palmitoyltransferase (SPT) catalyzes the condensation of palmitoyl-CoA and l-serine. The product of this reaction, 3-ketosphinganine (3KS), is further NADPH-dependently reduced to sphinganine (SphA) by 3-ketosphinganine reductase (3KSR). Ceramide synthases (CerS) couple fatty acyl-CoAs to the amino group of SphA. This leads to the formation of dihydroceramides (dhCer), differing in the chain length of the amide-bound fatty acid. The final step is the introduction of a double bond between carbons C-4 and C-5 mediated by dihydroceramide desaturase (DEGS) under NAD[P]H consumption. The formed ceramides (Cer) are subsequently shuttled to the Golgi apparatus via ceramide transporter (Cert) for further sphingomyelin synthesis. Parts of the image were taken from https://smart.servier.com, accessed on 1 May 2021 (freely accessible). (**b**) Visualization of sphingolipid metabolism in the ER via bio-orthogonal click chemistry using azidosphinganine and confocal laser scanning microscopy [27]. COS7 cells were transfected with (**A**) the ER-specific protein Sec61-mApple and incubated with (**B**) azidosphinganine and labelled with BODIPY-PEG_4_-DBCO. (**C**) Merged image showing good co-localization of both molecules in tubules of the ER. In contrast to Sec61, azidosphinganine also labelled homogenous structures between ER tubules which might represent ER sheets. Scale bar: 5 μm [27].

Sphingolipid de novo synthesis is initiated in endoplasmic reticulum (ER) by serine palmitoyl-transferase (SPT) catalyzed condensation of the activated C_16_ fatty acid palmitoyl-CoA and the amino acid l-serine to yield 3-ketosphinganine. This is then rapidly reduced to sphinganine by 3-ketosphinganine reductase (3KSR) in a NADPH-dependent manner. Then, sphinganine is further *N*-acylated by the action of six ceramide synthase isoforms (CerS1-6) encoded by six distinct genes to form ceramides [1]. The most notable characteristic of the individual CerS isoforms is a different acyl-CoA preference that can overlap within their isoforms. Variations of acyl-chain length in a cell type specific manner result in ceramide species with different biophysical properties and distinct biological functions. It has recently been possible to fully reconstitute and to monitor this de novo ceramide synthesis pathway in vitro by using both stably isotope labeled or bio-orthogonally modified precursors (Figure 1) [27,28].

**Figure 2:**
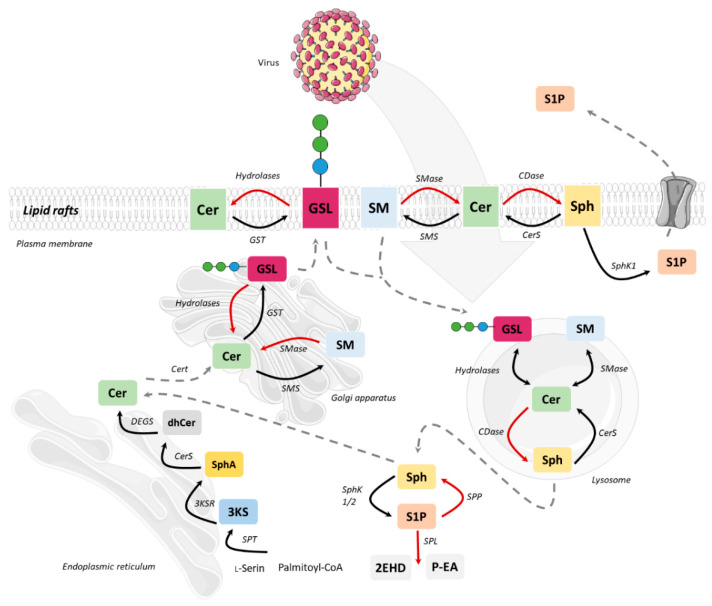
Schematic overview of the SL metabolism as potential target for viral interaction. After de novo synthesis in the ER, ceramide is transported to the Golgi apparatus via Cert. Ceramide is provided for the synthesis of complex sphingolipids such as sphingomyelin (SM) by sphingomyelin synthase (SMS) and glycosphingolipids (GSL) by glycosyl transferase (GST). Complex sphingolipids are transported from the Golgi apparatus to the plasma membrane or lysosomes. There the breakdown of SM to Cer takes place by sphingomyelinase (SMase), degradation of GSL occurs by the stepwise action of specific hydrolases. Subsequently, Cer can be degraded by ceramidase (CDase) to sphingosine (Sph). S1P can be formed by phosphorylation via sphingosine kinase (SphK1/2). Degradation of S1P occurs through S1P phosphatase (SPP) or S1P lyase (SPL) forming Sph or hexadecenal (2EHD) and phospho-ethanolamine (P-EA). Interaction with host cell membranous compartments is of key importance for replication of viruses and this therefore is highly dependent on SL metabolism.

Following transfer to the Golgi compartment, ceramides are further modified by glycosylation to yield glucosylceramides or acquisition of a phosphocholine headgroup via sphingomyelin synthase to yield sphingomyelin, both of which are transported to the plasma membrane using vesicular exocytosis where they, according to the topology of the modifying enzymes to the Golgi luminal compartment, predominantly localize to the outer membrane leaflet. As for other membrane lipids, trans-bilayer asymmetric distribution of SLs in the plasma membrane is regulated by the activity of scramblases at the steady state, or actively, by ATP-dependent flippases or floppases, and this is crucial for membrane integrity, charge and compartmentalization (reviewed in [29]). SL catabolism is initiated by ceramide production through the activity of sphingomyelinases or in the case of glycosphingolipids (GSLs) via specific hydrolases. Sphingomyelinases, depending on their pH optima, are grouped into neutral, acid and alkaline isoforms. Best studied amongst those include the neutral sphingomyelinase 2 (NSM2) which resides in association with the cytosolic membrane leaflet of the plasma membrane, multi-vesicular body, or the Golgi compartment, and is activated in response to a variety of signals including stress, cytokines or T cell receptor ligation [30,31,32,33]. Equally well investigated, acid sphingomyelinase 1 (ASM) anchors to anti-cytosolic membrane leaflets in the late endosomal compartment from where it is either secreted (soluble, sASM) or displayed after fusion with the plasma membrane in response to receptor signaling or during exocytic membrane repair [13,34]. Ceramide levels are tightly controlled as is reflected by its rapid conversion into ceramide 1-phosphate or de-acylation by ceramidases into sphingosine which, accumulating to even lower levels, serves as target for phosphorylation by sphingosine kinases to yield sphingosine 1-phosphate (S1P), a potent signaling molecule. Reflecting the dynamic demands to the system, anabolic and catabolic steps in SL metabolism are reversible except for the final breakdown of S1P into phospho-ethanolamine and hexadecenal by S1P lyase which thereby terminates the SL life cycle (reviewed in [2,35]). Notably, its phosphorylation renders S1P sufficiently polar and thereby soluble in the cytosol, from where it is also exported to initiate autocrine and paracrine S1P receptor signaling (Figure 2) [36,37].

## 3. Sphingolipid Targets in Viral Life Cycles

As their amplification is strictly intracellular, the interaction of viruses with cellular membranes is a key component of their life cycle, and this also includes availability and composition of most prominent membrane lipids such as SLs. The role of steady state SL metabolism and that regulated by pathogens in their respective host cells has been studied in the more recent past (for a recent review see [38]). This has been greatly advanced by progress made in terms of high resolution quantitative analysis and microscopy (see above) and implementation of functionalized SLs, allowing the study of topological accumulation in cellular compartments in the context of viral infection (recently reviewed in [39]). As detailed below, whole cell lipidomic analyses have clearly documented that viruses effectively modulate the SL pool in their host cells. It is, however, only upon implementation of this advanced overall methodical portfolio that the role of SLs (and eventually its individual metabolites) for defined steps within the viral replication process is understood at a cellular level. Furthermore, the availability of novel genetically modified cell, tissue and animal models continues to be instrumental for the understanding of the role of SL metabolism in viral pathogenesis which includes both viral replication per se, but also host responses, especially composition and function of the immune compartment. This knowledge will obviously be indispensable to identify and optimize SL targets for therapeutic purposes. This review will focus on examples where a role of SLs and their metabolization have been attributed to specific steps in the viral life cycle.

### 3.1. Attachement and Entry

Firm attachment to and passage of the host cell plasma membrane effectively initiate and thereby enable viral replication and therefore, the role of SLs in these processes has most intensely been studied. In particular, the role of membrane microdomains enriched for particular SL species and the impact of their accumulation or metabolization there has been studied with regard to receptor segregation, membrane biophysical alterations (favoring fusion or endocytosis) as well as initiation of signaling cascades involved in uptake and trafficking.

#### 3.1.1. Glycosphingolipids in Viral Entry

**Figure 3:**
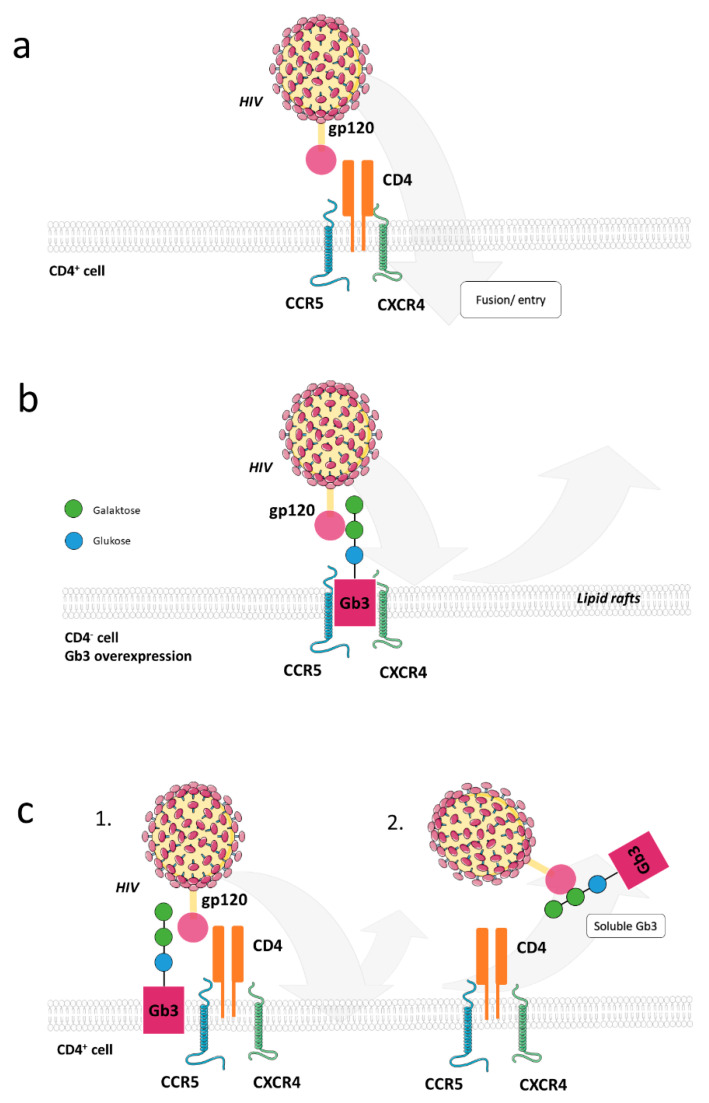
Schematic view of Gb3 interaction with HIV. (**a**) HIV infection requires gp120 binding to CD4. The associated conformational change of gp120 and simultaneous binding to chemokine co-receptors such as CXCR4 or CCR5 initiates cell fusion via gp41. (**b**) Gb3 overexpression in CD4-negative cells is associated with resistance to HIV infection as Gb3 is able to bind directly to gp120 and, consequently, block the chemokine binding motif. (**c**) Since T cells usually contain low levels of Gb3, HIV resistance is observed especially under conditions where Gb3 is overexpressed (1). In addition, soluble Gb3 analogues can bind to HIV gp120 and prevent binding to the CD4 receptor and the chemokine co-receptors, respectively (2).

Membrane domains enriched in GSLs (often also referred to as lipid rafts), are known as functional entities involved in cellular signal initiation, and may directly support viral host cell entry (Figure 3). Amongst the GSLs involved, Gb3 (globo-triasyl-ceramide) and galactosyl-ceramide (Gal-Cer), were found to interact with the V3 loop within HIV gp120 or the HIV gp41 envelope protein subunit, respectively, to facilitate their interaction with chemokine receptors and to support HIV uptake into CD4-negative cells such as mucosal epithelial cells. These then transcytose and transmit HIV to CD4+ T cells or DCs [40,41,42,43,44,45,46,47]. The importance of GSLs in HIV entry is further supported by its sensitivity to compounds affecting GSL biosynthesis such as D-threo-1-phenyl-2-decanoylamino-3-morpholino-1-propanol (PDMP), which inhibits glucosyltransferase activity [48], and to variations in cellular GSL content [49]. Interestingly, Gb3, when accumulating to high levels (for instance in PBMCs of Fabry disease patients or certain cell lines) can also act as a resistance factor for HIV infection [50,51,52,53]. Possibly, differential effects of Gb3 on HIV entry rely on its turnover, compartmentalization to membrane microdomains, and accessibility, all of which might be dependent on the respective host cell (Figure 3).

**Figure 4:**
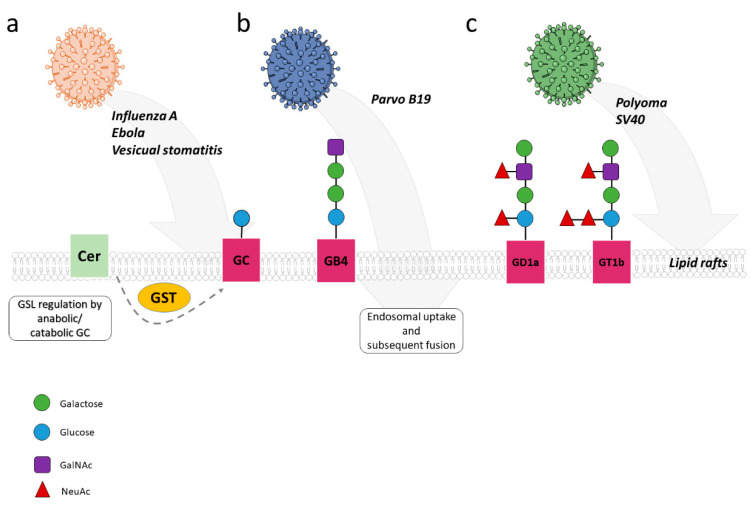
Influence of GSL on virus fusion and entry. Membrane domains enriched in glycosphingolipids (GSLs) are known to potentially directly support viral entry into host cells. (**a**) Glucosylceramide (GC) levels are particularly important for endosomal uptake of influenza A virus (IAV), Ebola, and vesicular stomatitis virus, whose uptake is sensitive to depletion of both anabolic and catabolic GC enzymes. (**b**) In addition, rearrangement of the viral parvovirus B19 capsid occurs through interaction with membrane-bound Gb4Cer (globo-tetraosyl-ceramide), which is required for subsequent steps of internalization in cells. (**c**) Gangliosides such as GD1a and GT1b or GM1 serve as important components in cell entry of murine polyomavirus and SV40. Notably, the interaction of SV40 VP1 with its cell surface ganglioside receptor GM1 is essential as a molecular trigger for SV40-induced vacuolization. Moreover, binding of gangliosides to 4-integrin promotes endocytosis and microtubular transport of polyoma virus capsids by initiating PI3K, FAK/Src, and MAPK pathways.

GSLs also effectively support entry of viruses other than HIV. Glucosylceramide (GC) levels proved to be particularly important in regulating uptake of viruses which rely on trafficking to and using the late endosomal compartment for fusion (Figure 4). These include influenza A virus (IAV), Ebola, SARS-CoV-2 and vesicular stomatitis virus entry of which was sensitive to depletion of both anabolic and catabolic GC enzymes [54,55,56].

Interaction with Gb4Cer (globo-tetraosyl-ceramide) triggers parvovirus B19 viral capsid rearrangements required for subsequent steps in internalization into cells also expressing the erythropoietin receptor [57]. Gangliosides (glycosphingolipids with one or more sialic acid residues linked to the sugar moiety) such as GD1a and GT1b or GM1 serve as important components in cell entry of murine polyoma virus and SV40, respectively (reviewed in [58]). Most notably, the interaction of SV40 VP1 with its cell surface ganglioside receptor GM1 has recently been identified as a molecular trigger for vacuolization caused by SV40 (which has led to its discovery). SV40, though not of great medical importance, was an invaluable tool to study principles of DNA replication and tumor biology in the past [59]. In line with the hypothesis that GSLs (as other sphingolipids, see below) support viral uptake by organizing membrane domains segregating membrane and membrane proximal proteins important in this process, they also perform the simultaneous engagement of gangliosides and α4-integrin promoted endocytosis and microtubular trafficking of polyoma viral capsids by initiating PI3K, FAK/Src and MAPK pathways [60]. Obviously, GSLs can be of crucial importance in uptake of certain viruses and therefore, interference with GSL biosynthesis might represent an interesting therapeutic option which has recently been excellently reviewed [61].

#### 3.1.2. Ceramide-Enriched Membrane Microdomains in Viral Uptake and Trafficking

Based on their biophysical properties, ceramide- enriched membrane domains which condense into larger platforms in response to sphingomyelinase activation or ceramidase inhibition, respectively, are sites of endocytic uptake of pathogens [6,62,63,64,65,66]. In addition to biophysical alterations promoting membrane vesiculation and fusogenicity [67], concentration of pathogen receptors and membrane proximal signaling complexes is believed to aid in pathogen uptake as indicated for GSLs above. Therefore, conditions favoring the generation of these domains (mainly by activation of sphingomyelinases or inhibition of ceramidase especially by inflammatory signals or viruses themselves on receptor interaction) would create a favorable environment enhancing viral infection.

This has in fact been verified for several viruses. For instance, the ability of CD300lf to support murine Norovirus entry was found to depend on SL biosynthesis, and more specifically, on ceramide generation. Thus, exogenous addition of ceramide restored susceptibility of serine palmitoyl-transferase deficient cells, and this relied on both formation of ceramide-enriched membrane domains and ceramide induced conformational changes of surface resident CD300lf proteins [68]. Sphingomyelinase activation also supported pH and clathrin-dependent entry and replication of Japanese encephalitis virus (JEV) in tissue culture, though the role of this enzyme in either process and its relevance in vivo has not been further analyzed [69]. Strikingly, SMS-1 generated SM rather proved to be important in Japanese encephalitis virus attachment and subsequent infection, and attenuation of JEV infection in SMS-1 deficient mice supported a pro-viral activity of SM in vivo [70].

Formation of ceramide-enriched platforms supporting entry into target cells by viruses has first been described for major and minor subgroup picornavirus rhinovirus (RV). RV attachment to epithelial cells initiated microtubule- and microfilament-dependent ASM cell surface translocation with subsequent formation of membrane domains enriched in ceramides or GSLs. Both SLs were found to interact with viruses and to be important in their uptake [71,72]. Intracellular RV trafficking involved particularly GSLs which were co-detected with endocytosed virus in membrane proximal and perinuclear vesicles. Interestingly, RV raft interaction also promoted biphasic activation p38 MAPK in a RhoA-dependent manner, with late activation relying on viral replication [73]. In addition to SM, ASM activity was also implicated in early steps of Ebola virus infection. While SM was required for attachment, viral particles strongly associated with surface displayed ASM, indicating that viral interaction may occur in SM-enriched membrane domains followed by ASM activation [74]. As for RV, receptors involved in ASM activation were not identified, and it also remained unclear whether ASM activation would be important in Ebola virus endocytosis and thereby, rendering the endo/lysosomal cholesterol transporter Niemann-Pick C protein 1 (NPC1) accessible to the viral particle. NPC1 was identified as crucial for Ebola virus uptake by enabling fusion between viral and endosomal membranes [75,76]. NPC1 acts as a bone fide receptor for the proteolytically activated viral envelope protein in an intracellular compartment rather than at the plasma membrane, and its activity ensures viral access to the cytoplasm [77].

**Figure 5:**
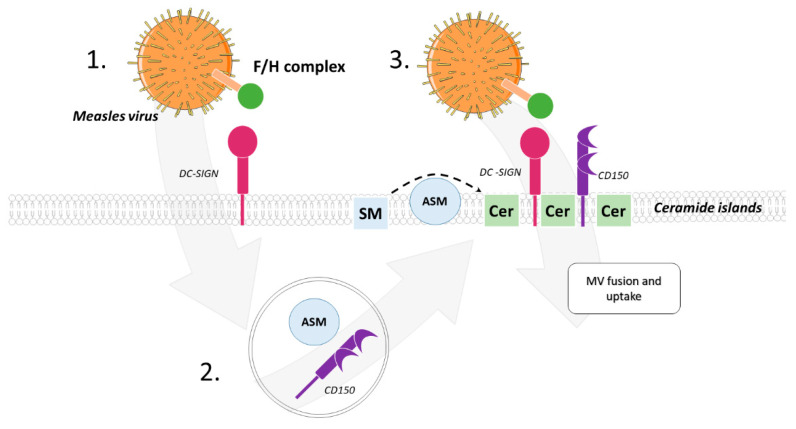
Ceramide-enriched membrane domains in MV infection. Interaction of MV glycoproteins with DC-SIGN on the DC surface activates translocation of ASM along with CD150 from intracellular endosomes to the cell surface. This makes the CD150, the MV entry receptor on hematopoetic cells, available to promote viral infection of DCs. Thus, activation of ASM subsequently promotes ceramide release in the membrane, making ASM activation a promising target in measles infection.

Promotion of viral entry by ASM activation through host cell surface interaction was mechanistically investigated for the measles virus (MV, an enveloped virus) in dendritic cells (DCs) [78] and for adenovirus (a non-enveloped virus) in epithelial cells [79]. Interaction of MV glycoproteins with DC-SIGN on the DC surface catalyzed activation of the ASM (and to some extent the NSM2) and subsequent ceramide release. Interestingly, this was also promoted by DC-SIGN ligation by specific antibodies or mannan revealing that this reflected DC-SIGN signaling per se and was not MV-specific. Along with ASM, CD150 (the receptor required for MV entry) translocated to the cell surface from an intracellular storage compartment and thereby, was made available to promote MV infection of DCs. Whether or not CD150 surface translocation on DCs may be important for pathogens other than MV has not been investigated. On murine macrophages CD150 can, however, also serve as a microbial sensor routing Gram-negative bacteria into phagocytic compartments [80] (Figure 5).

Interaction with its surface receptors CAR and α3 integrin causes limited uncoating of the non-enveloped adenovirus particle at the cell surface which leads to exposure of the adenoviral membrane lytic protein-IV. Similar as described for bacterial toxins [81,82,83], this protein causes membrane lesions followed by Ca^2+^-influx promoting a wound repair process by subsequent lysosomal exocytosis along with ASM surface display and formation of ceramide-enriched membrane domains [79]. These act to enhance viral endocytosis and to recruit and concentrate lytic protein-VI in endosomes, thereby catalyzing endosomal leakiness and finally rupturing as required for release of the viral capsid into the cytosol. In this study, ceramide release and concentration within endosomes has clearly been revealed as crucial for protein-IV recruitment and subsequent viral release from these compartments. Most interestingly, a recent study identified sphingosine accumulation as a result of ceramide breakdown by acid ceramidase as a cell intrinsic antiviral defense mechanism in macrophages. In these cells, herpes simplex virus (HSV-1) was found trapped in endosomal compartments enriched for sphingosine, and ablation of acid ceramidase promoted HSV-1 capsid export into the cytosol [84]. Acid ceramidase expression was induced downstream of IRF-8 signaling and in this model, sphingosine production proved to be the crucial effector for protection from infection in vitro and in vivo.

The recent SARS-CoV-2 pandemic has initiated an intensive search for pre-emptive and therapeutic approaches, also including studies on drug re-purposing. As for many other viruses before, the integrity of lipid rafts has proven to be of crucial importance in SARS-CoV-2 entry both at the level of fusion at the plasma membrane and endocytosis-mediated uptake. Consequently, lipid raft targeting drugs were effective at preventing SARS-CoV-2 entry into a variety of host cells in vitro (reviewed in [85]). In particular, ASM activity as induced after SARS-CoV-2 binding to its receptor [86,87] has been identified as a promising target for intervention.

**Figure 6:**
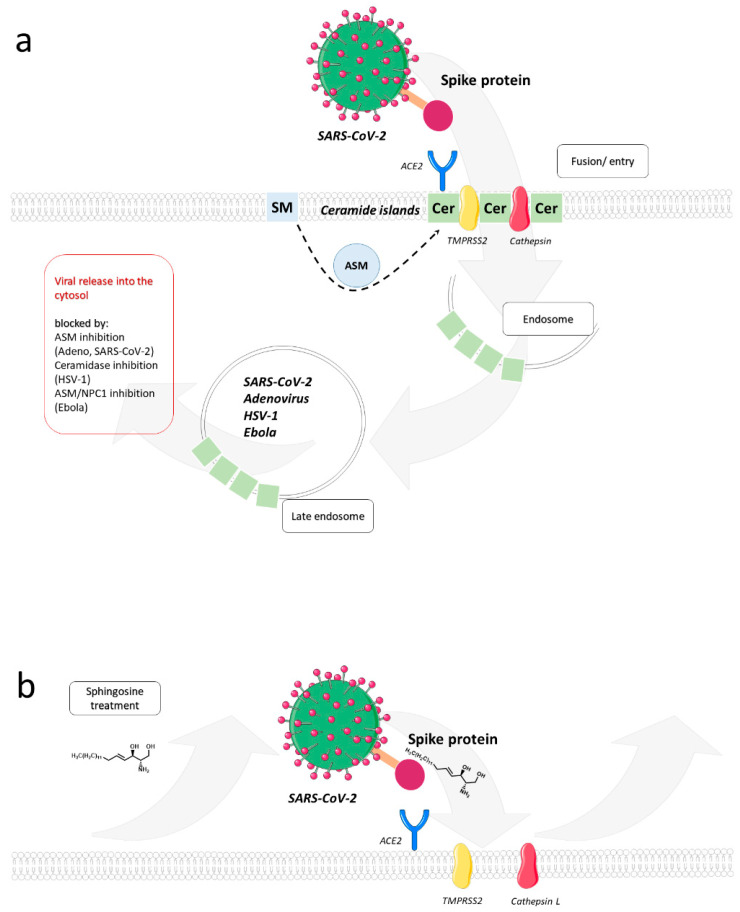
Ceramide-enriched membrane domains in SARS-CoV-2 infection. (**a**) To enter host cells, SARS-CoV-2 first binds to a cell surface receptor and then enters endosomes. The spike protein, located on the viral surface, mediates coronavirus entry via the receptor-binding domain (RBD), which specifically recognizes angiotensin-converting enzyme 2 (ACE2) as its receptor. In addition, the SARS-CoV spike protein must be proteolytically activated for membrane fusion by proteases such as TMPRSS2 or the lysosomal proteases cathepsins, so it undergoes a dramatic structural change. Ceramide-enriched membrane domains, which condense into larger platforms in response to sphingomyelinase activation or ceramidase inhibition, are sites for endocytic uptake of SARS-CoV-2. Drugs that inhibit ceramide biosynthesis have been effective against SARS-CoV-2 entry into host cells. In particular, ASM activity induced after binding of SARS-CoV-2 to ACE-2 is a promising target in SARS-CoV-2 infection. In common with that of other viruses, release of virus from endocytic compartments relies on ceramide metabolism. (**b**) Application of exogenous sphingosine could prevent interaction with SARS-CoV-2 spike protein and thereby exert antiviral activity.

Fluoxetine, but not two other serotonin uptake inhibitors tested, escitalopram and paroxetine, inhibited SARS-CoV-2 replication in Vero cells by more than two log, indicating that the inhibitory activity of these compounds did not segregate with serotonin uptake inhibition [88]. The activity of fluoxetine as functional inhibitor of ASM activity (FIASMA) might be required to target early steps of SARS-CoV-2 host cell interaction as supported by two further studies. Both genetic and pharmacological ASM inhibition (the latter exerted by a variety of compounds also including fluoxetine and escitalopram) reduced SARS-CoV-2 infection in several cell lines and primary nasal epithelial cells by preventing formation of ceramide-enriched membrane platforms required for viral uptake [89]. It appeared that ASM activity and ceramide release were promoted by host cell interaction by the viral S protein with its receptor, ACE-2 [86,87,89]. In addition, focusing on fluoxetine, a third group confirmed inhibition of SARS-CoV-2 by this compound, providing solid evidence that accumulation of cholesterol and pH buffering downstream of ASM inhibition in late endosomal compartments prevented fusion of viral and late endosomal membranes as required for viral uncoating [90]. In line with the importance of cholesterol homeostasis and acidification in late endosomes (both targeted by fluoxetine) being crucial for SARS-CoV-2 replication, both a cholesterol efflux inhibitor and pH buffering (most likely preventing the activity of proteases required for the release of the coronaviral fusogenic peptide within the S protein) were effective at inhibiting virus production from Vero and Calu-3 cells. Interaction of the viral spike protein with its receptor-binding domain of ACE2 was found to be prevented upon exogenous supply of sphingosine, which directly associated with this receptor [91]. Thus, sphingosine, known for its bacteriocidal activities in the respiratory tract (recently reviewed in [92,93]), might also exert antiviral activities at the level of entry in this particular compartment (Figure 6).

#### 3.1.3. Antiviral Activity of Ceramide at the Level of Uptake

Ceramide release may, however, also act antivirally at the level of uptake. Entry of HIV into T cells, monocytes or macrophages was highly sensitive to compounds elevating levels of ceramides such as exogenous addition of long chain ceramide (C_16_) which prevented lateral diffusion of CD4 towards the chemokine co-receptors [94,95,96]. For optimal HIV entry, gp41-mediated membrane fusion was found to be rather dependent on sphingomyelin synthase-2 (SMS2) activity indicating that SM rather than ceramide accumulation was important in this process [97]. Similarly, overall elevation of ceramides by bacterial sphingomyelinase interfered with uptake of hepatitis C virus (HCV) at the level of receptor segregation. CD81, a major entry factor, was partially internalized, and this and other components required for HCV entry, scavenger receptor B1 and claudin-1, were excluded from detergent resistant microdomains [98].

Actin dynamics is important for uptake of most if not all viruses. This includes, for example, drifting and surfing of receptors engaged by viruses along filopodia or on the cell body surface [99,100,101], receptor clustering, and formation of and transmission by defined structures such as virologic synapses or filopodial bridges [102,103]. Moreover, actin-mediated membrane ruffling and blebbing is essential for macro-pinocytic uptake of, for instance, vaccinia, picorna and adenoviruses [104,105]. Thus, interference with actin dynamics would be expected to have a significant impact on uptake efficiency. In this context it is noteworthy that breakdown of actin cytoskeletal protrusions after ASM activation and subsequent ceramide accumulation were observed in MCF-7 breast cancer cells [106], and NSM- and ASM-dependently, upon measles virus (MV) interaction with T cells [107,108].

### 3.2. Regulation of Viral Replication via Sphingolipids

When evaluated at an overall level, individual SL species accumulating in viral host cells may be favorable for either the host cell or the virus. Lipidomic profiling in a bronchial epithelial cell line revealed massive perturbation of particularly the SL pathway, and therein, most prominently in sphingomyelin species, after infection by influenza A virus, rhinovirus and SARS-CoV-2. Revealing that requirements of sphingomyelin in viral replication might substantially differ, exposure of infected cells to bacterial sphingomyelinase suppressed replication of influenza virus and SARS-CoV-2, yet enhanced that of rhinovirus [109]. Additionally, in lung epithelial cells, a protective role of ceramides was evidenced where replicating, but not UV-inactivated IAV caused de novo biosynthesis of ceramide which limited viral replication [110] as previously also suggested for hepatitis B [111]. In contrast, SLs, also including ceramides, may also act pro-virally by supporting viral replication as revealed for hepatitis C, West Nile and Dengue viruses [112,113,114].

These are positive strand RNA viruses known to extensively remodel cellular membranes into distinct compartments referred to as viral replication compartments (VRCs). VRCs act as platforms to concentrate viral proteins and assemble replication complexes, and shield those from recognition by innate defense systems. Obviously, virally-triggered rewiring of host lipid metabolism creating a specific lipid micromilieu is essential in VRC formation and function. This has been extensively studied for sterols and glycerophospholipids (excellently reviewed in [115]) while the role of SLs in this process is less well understood. Pharmacological inhibition of SL biosynthesis interfered with replication of hepatitis C and West Nile Virus, and SM, glycosphingolipids or ceramide, respectively, were detected in association with VRCs [116,117,118]. Mosquitos, if infected by an intracellular bacterial genus called Wolbachia, are much less efficient at transmitting Dengue Virus to humans. Supporting a pro-viral role of SLs in Dengue virus replication, all SL classes found to be enriched in Dengue infected mosquito cells were depleted in the presence of Wolbachia, which obviously created an unfavorable lipid environment to the virus [119]. A comprehensive lipidomic study recently revealed significantly remodeled lipid composition in Huh7 cells upon infection with Zika virus (a flavivirus) (or ectopic expression of its nonstructural protein 4B (NS4B)) and this particularly affected SLs subclasses [120]. SLs were found to act pro-virally for Zika virus infection, because inhibition of SL biosynthesis interfered with viral replication in a variety of cell types while exogenous supply of ceramide sensitized target cells for viral infection. Interestingly, ceramide was found to redistribute to Zika virus replication sites associating with NS4B, there suggesting that ceramide flux takes part in VRC formation and activity.

Regulation of the sphingosine kinase/sphingosine-1-phosphate system by viruses has been widely studied [121] and proposed as a potential target for intervention in viral infections, also including COVID-19 [122]. It can act antivirally as revealed for viral diarrhea virus (BVDV), a close relative of HCV. A nonstructural BVDV protein (NS3) binds to and inhibits sphingosine kinase 1 (SphK1) and this was found important for efficient viral replication [123]. Similarly, pharmacological activation of SphK1, overexpression of the enzyme or administration of S1P, effectively interfered with Ebola virus glycoprotein driven entry into endothelial cells [124]. In contrast, activation of neutral ceramidase and SphK1 and as a result of sphingosine 1-phosphate (S1P) generation, AKT and ERK supported replication of respiratory syncytial virus (RSV) in lung epithelial cells [125]. A pro-viral role of sphingosine kinase/S1P was also seen for IAV, MV and human cytomegalovirus replication (HCMV) [126,127,128]. Thus, SphK1 overexpression enhanced IAV protein synthesis and progeny virus synthesis [129], while SphK1 inhibition reduced viral replication by interfering with nuclear export of viral RNPs [130]. IAV infection itself activates SphK1 [129] and thereby stimulates the NFκB pathway which promotes viral RNA synthesis [131]. Similar observations were made for MV infection, where SphK inhibition impaired viral protein expression and suppressed MV-induced activation of NFκB in certain cell lines [127]. In its natural (lymphoid) target cells, however, inhibition of both acid ceramidase (causing ceramide accumulation) or sphingosine kinase (elevating sphingosine) impaired MV replication. Rather than acting on a viral target directly, the latter particularly affected cellular activities including mTORC1 and Hsp90, supporting the interpretation that MV promotes activation of these pathways to ensure efficient replication [132]. Recent studies performed in 3D cultures modeling the respiratory tract supported a key role of MV-induced S1P to promote fast ameboid migration of DCs toward the lung epithelial cell layer as important for transmission during viral exit from the infected individual [133].

### 3.3. Sphingolipids in Viral Assembly, Maturation and Infectivity

The membrane patch where viral assembly occurs defines the composition of the viral particle’s envelope membrane. Initially established as highly relevant in membrane model systems, the operation of lipid-based protein sorting in mammalian cell membranes has in fact been pioneered by studies on HIV biogenesis [134,135]. When comparatively analyzed with that of the overall cellular membrane, the HIV particle substantially differs in its lipid composition as especially reflected by selective enrichment of SM and dihydro-sphingomyelin, while ceramides are barely represented [136,137]. This suggested that the viral core either selects already existing or actively remodels host cell membranes during the assembly and subsequent budding process. It was only after techniques to visualize and trace single virus assembly by quantitative live cell imaging that, driven by oligomerized HIV Gag protein at the inner membrane leaflet, formation of ordered membrane domains orchestrating lipid and protein composition could be demonstrated [135,138]. Underlying mechanisms described in this and earlier highly elegant studies included acquisition of membrane curvature [139,140], lipid-based phase partitioning and sequential sorting of proteins, and supported the importance of trans-bilayer coupling by acyl chain interactions for phase separation of the outer membrane leaflet assembly site.

As indicated above for VRCs, biogenesis of lipid structures can be intimately coupled to viral replication and/or assembly for certain viruses. The latter process has been intensely studied for HCV and Dengue virus, where biogenesis of lipid droplets has a crucial role in initiation of viral assembly [141,142]. Interestingly, ceramide transfer protein (CERT) was required for HCV maturation, suggesting an important contribution of the SL pathway in flavivirus replication [143].

Finally, the lipid composition of the enveloped viral particle may substantially impact its infectivity. Suggesting that SM content of the particle is important, viral entry was reduced after treatment of bovine herpesvirus particles, but not that of the target cells with bacterial sphingomyelinase (bSMase). In contrast, pseudorabies virus entry was sensitive to bSMAse at the level of the host cell and not the particle, while HSV-1 entry was insensitive to bSMase exposure of either membrane or particle [144] and SM was found to be important in IAV infection both at the viral particle and the host cell level [145]. As especially revealed for HIV and Ebola virus particles, viral uptake into DCs is substantially enhanced upon recognition of sialylated gangliosides anchored to viral membranes by Siglec-1 [146,147].

Infectivity of viral particles budding into intracellular compartments may also be determined by their SL composition. Thus, the HCV RNA-dependent polymerase NS5B and p7 protein cooperatively promote infectivity of the viral particle by decreasing its SM content [148], and morphogenesis of BVDV, budding into the ER, also includes a lipid sorting mechanism [149]. Viral particles were found particularly enriched in cholesterol, SM and hexosyl-ceramide, with both cholesterol and SM being of functional importance in attachment and entry of BVDV.

## 4. Outlook and Perspectives

Drug repurposing has already revealed the potential but also limitations of SL pathway modulating compounds in containing viral infections. This has so far been studied for the SphK/S1P/S1P lyase system and results obtained clearly document the complexity of responses. FTY720 (commercially known as fingolimod or Gilenya), upon phosphorylation by SphK2, acts to inhibit S1P receptor signaling, and thereby, potently inflammation and is therefore licensed for treatment of multiple sclerosis. A recent study suggested fingolimod as a promising novel therapy approach for HIV treatment and prevention. Firstly, it prevented viral spread in human CD4+ T cells by reducing surface density of this receptor (which may be of particular relevance given the low density of HIV glycoproteins available for interaction), and secondly, it enhanced the activity of SAMHD1, a cellular restriction factor, and thereby, levels of total and integrated HIV [150]. Thus, in addition to preventing S1P-mediated cellular activation which overall is mainly beneficial for viral replication, fingolimod has apparently virus-specific targets as well. Although it did not mechanistically address the role of S1P or S1PR signaling, a recent study provided compelling evidence that SphK2, the enzyme required to activate fingolimod, efficiently prevented clearance of LCMV in experimentally infected mice by restricting T cell immunopathology and promoting viral persistence [151]. Based on its anti-inflammatory activity, fingolimod (or, in general terms, inhibitors of S1P receptor signaling) has also been evaluated for its beneficial effect in virally-induced immunopathology when brought about by hyperinflammation through the activity of immune effector cells or cytokine storm. Thus, S1P analogs effectively reduced lung pathology induced by experimental influenza or paramyxovirus infection in mice [152,153,154]. Cytokine storm and hyperinflammation associated with prominent lung infiltration of CD8+ T cells and NK cells also marks the late phase of SARS-CoV-2 infection in mice, and therefore, S1P analogs in COVID-19 therapy might be an option [122]. This needs, however, to be critically assessed because interference with S1PR signaling obviously also limits the ability of the host to clear viral infection by both disabling recruitment and activation of effector cells and by limiting IFN-responses [152,155]. Interestingly, a host-protective role has been revealed for the S1P lyase in vitro; independently of its ability to catalyze S1P this enzyme enhanced type I interferon production in influenza virus infection [156].

Another example where targeted intervention of the SL pathway may be highly effective in viral pathogenesis is production of extracellular vesicles (EV). These come in different types, dependent on their origin, and production of least some EV species was found to occur clearly ASM or NSM-dependently [157,158,159,160]. Viral infections are known to affect generation and content of EVs, and thereby mediate transfer of both viral and host cell proteins or nucleic acids [161]. Viral components transferred by this pathway include single proteins, sub-genomic or micro-RNAs, up to entire viral particles, the latter revealed for hepatitis C and A viruses. Alternatively, EVs transfer cellular or viral proteins involved in regulating the interferon response of yet uninfected neighboring cells. For instance, EVs were found to amplify the innate immunity to hepatitis B, mouse hepatitis and adenovirus in liver cells in vitro and in vivo by transfer of interferon effector proteins from infected cells to uninfected cells, and importantly, this was prevented by both pharmacologic inhibition and genetic ablation of NSM2 (in vivo mediated by hydrodynamic injection of siRNA) [162]. Targeting the activity of SL metabolizing enzymes involved in EV production might thus be highly efficient in regulating viral pathogenesis at multiple levels. 

Apparently, yet not surprisingly, the impact of the SL anabolic and catabolic pathway on viral replication occurs at multiple levels, and doubtlessly, much more will be detected in the future now that experimental systems have advanced. This especially refers to mouse models allowing for—ideally conditional—genetic ablation of enzymes involved in the SL pathway, in particular compartments that will be of particular relevance to study its role in viral pathogenesis and to evaluate the potential of pharmacologic intervention. It is, however, essentially clear that interventions into viral replication per se are highly effective when implemented early in infection, and thereby need to be complemented by drugs modulating induction and activity of host immune responses.

## Data Availability

Not applicable.
